# Effects of 3 days of citrulline malate supplementation on short‐duration repeated sprint running performance in male team sport athletes

**DOI:** 10.1002/ejsc.12090

**Published:** 2024-03-18

**Authors:** Vinicius S. Faria, Brendan Egan

**Affiliations:** ^1^ School of Health and Human Performance Dublin City University Dublin Ireland; ^2^ Florida Institute of Human and Machine Cognition Pensacola Florida USA

**Keywords:** ergogenic aid, lactate, maximal shuttle run test, performance decrement

## Abstract

Citrulline malate (CM) is purported to be an ergogenic aid during various types of exercise performance. However, the effects of CM on repeated sprint performance (RSP) are under‐explored. In a placebo‐controlled, double‐blind, counterbalanced cross‐over design, male university‐level team sport athletes (*n* = 13) performed two familiarization trials, after which CM or placebo (PLA) (8 × 1 g tablets each day) were taken on the 2 days prior to, and with breakfast on the morning of, each main experimental trial. The main experimental trials employed a RSP protocol consisting of 10 repetitions of 40 m maximal shuttle run test (MST) with a 30 s interval between the start of each sprint. Sprint times and heart rate were recorded throughout the MST, and blood lactate concentrations were measured before, immediately after, and 5 min after completing the MST. CM resulted in better RSP compared to PLA, as indicated by a lower sprint performance decrement (S_dec_: CM, 4.68% ± 1.82% vs. PLA, 6.10% ± 1.83%; *p* = 0.03; ES = 0.77), which was possibly influenced by the fastest sprint time being faster in CM (CM, 8.16 ± 0.34 s vs. PLA, 8.29 ± 0.39 s; *p* = 0.011; ES = 0.34). There were no differences between CM and PLA in average sprint time (*p* = 0.54), slowest sprint time (*p* = 0.48), blood lactate concentrations (*p* = 0.73) or heart rate (*p* = 0.18), nor was there a condition × time interaction effect across the 10 sprints (*p* = 0.166). Three days of CM supplementation (8 g daily) attenuated the sprint performance decrement during short‐duration high‐intensity exercise in the form of running RSP in male university‐level team sport athletes.

## Highlights

- This study addresses a knowledge gap around the ergogenic potential of citrulline malate (CM) in repeated sprint running performance.
- Three days of CM supplementation (8 g daily) was observed to improve short‐duration (5 min) high‐intensity exercise performance in male university‐level team sport athletes when measured by the sprint performance decrement in a 10 repetition 40 m maximal shuttle run test.
- The attenuation in the sprint performance decrement was not associated with any differences compared to the placebo condition in heart rate or blood lactate concentrations.
- The outcomes of this study suggest that short‐term CM supplementation could be beneficial for team sport athletes who perform short‐duration high‐intensity efforts, such as soccer, Gaelic games, field hockey, and rugby.

## 1 INTRODUCTION

Citrulline malate (CM) is an organic salt formed through the combination of L‐citrulline (C_6_H_13_N_3_O_3_) and malate (or malic acid, C_4_H_6_O_5_) (Gough et al., 2021). L‐citrulline is commonly known as a non‐essential amino acid involved in the urea cycle, while malate is a tricarboxylic acid (TCA) cycle intermediate. CM sold as a pharmaceutical agent (brand name: Stimol) was originally developed to enhance muscle performance and to mitigate muscle recovery time in humans with asthenia after acute disease (Creff, 1982; Dauverchain, 1982), for which the recommended dose was 1 g taken 3 times a day (Bendahan et al., 2002; Pérez‐Guisado & Jakeman, 2010).

The potential efficacy of CM as an ergogenic aid is based on three hypothetical mechanisms of action: (1) via the L‐arginine nitric oxide (NO) pathway, which may improve the delivery of blood (and oxygen) to and from the active musculature during exercise (Wax et al., 2015); (2) via ammonia detoxification through the urea cycle, which may decrease lactate production and enhance the erobic utilization of pyruvate, thereby improving muscle function and attenuating fatigue (Gonzalez & Trexler, 2020); and (3) via the malate component, which has been proposed to augment energy production and increase the rate of adenosine triphosphate (ATP) production (Bendahan et al., 2002; Gonzalez & Trexler, 2020). Additionally, CM supplementation is well‐tolerated, with studies using 12 g (Cunniffe et al., 2016), 8 g (Chappell et al., 2020, 2018; Farney et al., 2019; Glenn et al., 2016, 2017; Gonzalez et al., 2018; Pérez‐Guisado & Jakeman, 2010; Wax et al., 2015; Wax et al., 2016), 6 g (Da Silva et al., 2017; Figueroa et al., 2015), and 2 g (Hwang et al., 2018). As an ergogenic aid, the most frequently used dosage has been a single dose of 8 g consumed 1 h before the exercise session (Gonzalez & Trexler, 2020), yet the optimal dosing strategy remains to be established. Several studies have used from 1 to 16 days of supplementation, with these longer supplementation periods being associated with greater likelihood of a benefit to performance (Viribay et al., 2022).

L‐citrulline is a popular ingredient in multi‐ingredient pre‐workout supplement formulations (Jagim et al., 2019). For more than a decade, CM has been purported to be an ergogenic aid during various types of exercise, such as resistance exercise (Chappell et al., 2020, 2018; Da Silva et al., 2017; Farney et al., 2019; Glenn et al., 2016, 2017; Gonzalez et al., 2018; Hwang et al., 2018; Pérez‐Guisado & Jakeman, 2010; Trexler, Keith, et al., 2019; Wax et al., 2015, 2016), cycling (Cunniffe et al., 2016; Glenn et al., 2016), and running (Bezuglov et al., 2022). However, assessing physical fitness and performance in tests that are representative of the physiological demands of the sport is essential (Charron et al., 2020). The ability to perform submaximal or maximal repeated sprints interspersed with limited periods of recovery between sprint efforts, termed repeated sprint ability, is an important fitness component for performance in competitive team sports (Bishop et al., 2011; Charron et al., 2020; Spencer et al., 2005).

There are demonstrated benefits of CM supplementation on short‐duration performance in humans (Cunniffe et al., 2016; Glenn et al., 2017; Pérez‐Guisado & Jakeman, 2010; Wax et al., 2016), yet across the domains of strength, power, high intensity, and aerobic exercise performance, the findings are equivocal (Trexler, Persky, et al., 2019; Vårvik et al., 2021; Viribay et al., 2022). Moreover, the effects of CM supplementation on repeated sprint performance (RSP) in team sport athletes are under‐explored. Therefore, this study investigated the effect of 3 days of CM supplementation (8 g daily) on short‐duration high‐intensity exercise performance in the form of running RSP in male university‐level team sport athletes.

## 2 METHODS

### 2.1 Participants

Thirteen male athletes (*n* = 13; age, 21.3 ± 1.9 years; height, 1.82 ± 0.06 m; body mass, 78.6 ± 9.5 kg; BMI, 23.6 ± 1.8 kg.m^−2^) were recruited from university rugby union, soccer, Gaelic games, and field hockey teams. Inclusion criteria were to be male, aged 18–30 years, actively training and competing at least three times weekly in team sport at university level and a respective team outside of the university, and free from neuromuscular and musculoskeletal disorders and lower limb injuries for at least 6 months prior to the study. All testing was conducted during their competitive season. Participants were excluded if suffering from any pathology or injury, or using any medications for metabolic or cardiovascular conditions, or using the dietary supplements beta‐alanine or creatine, either in isolation or in pre‐workout supplements. Recruitment took place via advertisement posters erected on the university campus, and sharing of recruitment materials with coaches of the respective university teams. Twenty‐two participants expressed interest in participating in the study, four of whom were excluded for reasons of supplement use or injury history, and five who commenced but did not complete the study due to scheduling conflicts or inability to give the required time commitment. The University College Dublin Research Ethics Committee, in accordance with the Declaration of Helsinki, approved all experimental procedures (permit LS‐11‐156). Each participant provided written informed consent prior to participation.

### 2.2 Experimental design

**FIGURE 1:**
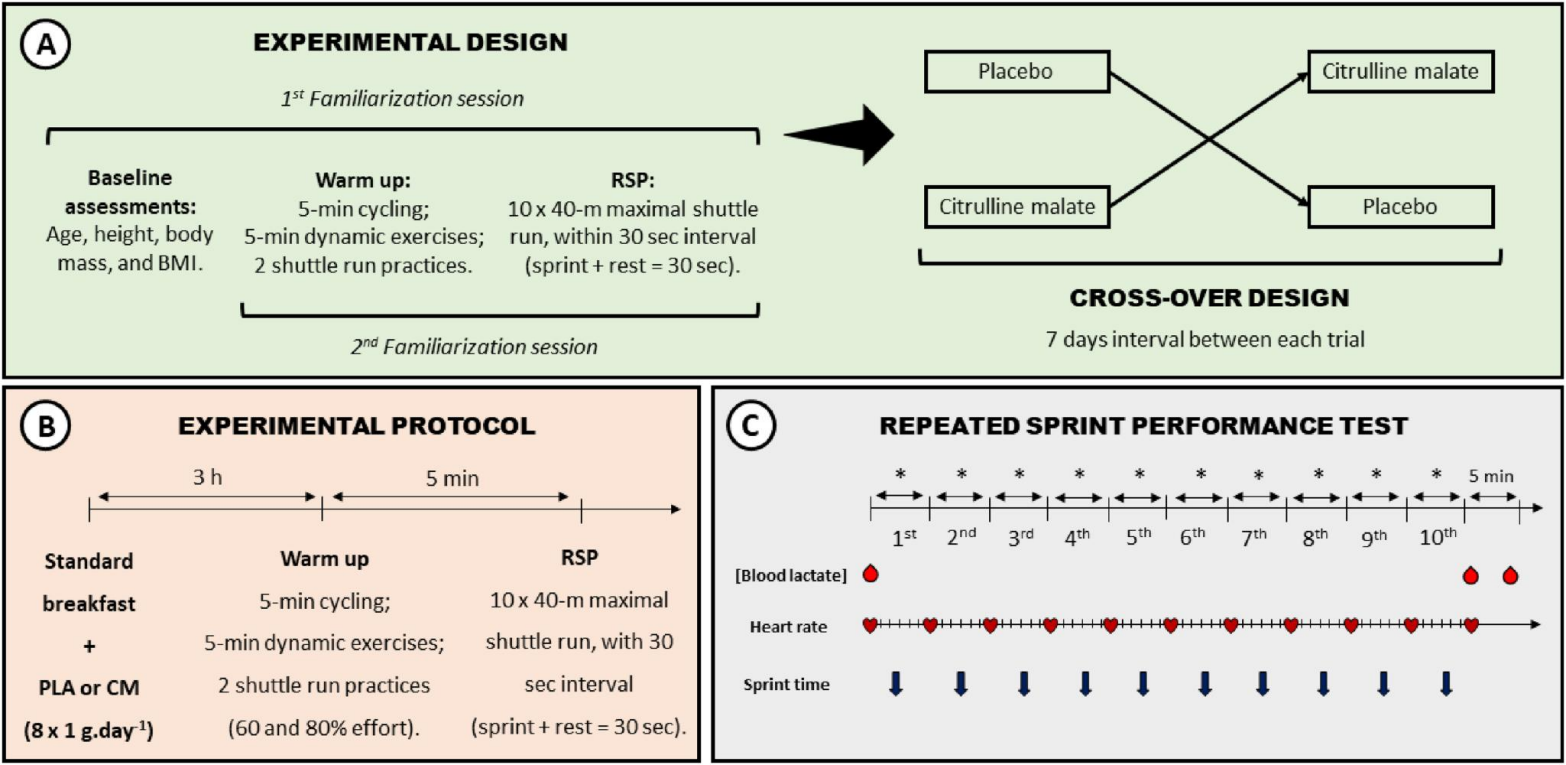
Timeline of events throughout the study. (A) Participants attended the laboratory for four visits. The first visit consisted of baseline assessments including age, height, body mass, and BMI, and familiarization with the RSP test; the second visit was a second familiarization with the RSP test; the third and fourth visits were the main experimental trials performed in a placebo‐controlled, double‐blind, cross‐over design of CM or PLA (8 g.day^−1^) for 3 days (i.e., the 2 days prior to, and the day of each trial). (B) For each experimental trial, 3 h after the last administration of either supplement with breakfast, and after a brief warm‐up, the participants undertook the RSP test. (C) The RSP test required participants to complete ten repetitions of 40 m maximal shuttle run test with a 30 s interval between the start of each sprint (*, recovery time between sprints was equivalent to 30 s minus the time taken to complete the previous sprint). Sprint times (↓) and heart rate (♥) were recorded throughout the RSP protocol, and blood lactate concentrations (●) were measured before, immediately after and 5 min after completing the RSP. BMI, body mass index; CM, citrulline malate; g, grams; h, hours; m, meters; min, minutes; PLA, placebo; RSP, repeated sprint performance; s, seconds.

In a placebo‐controlled, double‐blind cross‐over design, participants visited the High Performance Unit at University College Dublin on four separate testing occasions. The first two visits were to familiarize the participants with the study procedures and protocol for the 40 m maximal shuttle run test (MST) that was employed as the test of short‐duration RSP (Baker et al., 1993; Glaister et al., 2009). Two familiarization sessions are required in order to mitigate the learning effect and improve reliability of this protocol (Glaister et al., 2009). The familiarization sessions were followed by CM and placebo (PLA) trials with 1 week interval between each of the four visits. All sessions took place on the same indoor tartan running track in the same clothing and footwear, and were conducted at the same time of day ±1 h to reduce the influence of chronobiological variation (Figure 1A). Across all visits, ambient temperature ranged from 16 to 19°C, and relative humidity ranged from 63% to 83%. Noise levels were minimized, and music was not permitted.

For all visits, participants presented to the laboratory having consumed the same self‐selected breakfast 3 h before each trial. Shortly after arrival, a resting blood lactate concentration was measured from a finger prick sample (Lactate Pro2, Arkray). A standardized warm‐up was performed consisting of 5 min on a stationary cycle ergometer, followed by 5 min of supervised dynamic exercises (walking lunges, marching, heel flicks, high knees, leg swings). Prior to the MST, participants then performed two practice runs of the 40 m shuttle for which they were asked to perform at intensities of 60% and 80%, respectively, of their perceived maximum effort, with 1 minute rest taken between these efforts.

The MST protocol, as previously described (Baker et al., 1993; Glaister et al., 2009), required the participants to complete 10 repetitions of the 40 m shuttle with a 30 s interval between the start of each sprint (i.e., recovery time between sprints was equivalent to 30 s minus the time taken to complete the previous sprint) (Figure 1B). Participants ran between two lines placed 20 m apart with the start/finish line placed at the midpoint of the course. On instruction, each participant sprinted 10 m from the start/finish line to the end of the course, turned 180°, sprinted 20 m to the other end of the course, turned 180°, and sprinted 10 m back through the start/finish line. Each sprint was initiated from a line 50 cm behind the start line to prevent false triggering of the first timing gate. Participants were instructed to place at least one foot over the line at the turns of each shuttle, the adherence to which was monitored to ensure full compliance. For recovery between each sprint, participants walked slowly back to the start line and remained standing in preparation for the next sprint. Sprint times were recorded to the nearest 0.001 s using photoelectric timing gates (FusionSport) positioned at the start/finish line. Single‐beam speed gates have previously shown adequate reliability for the measurement of sprint performance (ICC = 0.87–0.96; CV = 1.4%) (Gabbett et al., 2008; Haugen et al., 2014). Heart rate was monitored by telemetry in 5 s intervals throughout the MST (Polar). A second and third blood lactate measurement was performed immediately (<30 s), and 5 min after, completion of the MST, respectively (Figure 1C). The Lactate Pro2 analyzer has a manufacturer‐reported CV of ∼3%, which is in broad agreement with published studies that is, 2.7%–4.3% at concentrations between 5.0 and 14.9 mM (Bonaventura et al., 2015) and 3.3% at concentrations >8.0 mM (Crotty et al., 2021). No feedback on sprint times was given to the participants during trials. Verbal encouragement to give maximum effort for each sprint was standardized and provided throughout each visit.

### 2.3 Pre‐trial preparation

All visits took place at least 72 h after any match performance in a participant's respective sport, and at least 36 h after the most recent training session. Participants were asked to abstain from alcohol consumption for at least 24 h before each visit, and asked to abstain from caffeine consumption on the morning of each visit. Prior to the first visit, participants were asked to keep a portion estimate food diary for the day before that visit, and for their breakfast on the morning of the coming visits. A copy of this food diary was provided back to participants in advance of subsequent visits for which they were asked to follow this exact pattern of intake in addition to consuming 500 mL of water with breakfast. Compliance with each of these pre‐trial standardization practices was confirmed verbally on arrival to the laboratory each day.

### 2.4 Administration of citrulline malate and placebo

Citrulline malate (CM; CM1000, ROS Nutrition) and PLA in the form of an essential amino acid mix (ISO EAA, ROS Nutrition) tablets were provided to participants by a researcher not involved in the MST. These main experimental trials were separated by 7 days, and performed in a counterbalanced manner. Eight tablets containing 1000 mg of CM or PLA each (8 × 1 g.day^−1^) were given to the participants for consumption with their breakfast for 3 days (i.e., the 2 days prior to, and on the day of the trial). Participants undertook the MST 3 h after the ingestion of either supplement. The PLA was chosen in order to be identical in size, format, color, taste, and texture to CM, and thereby make the conditions indistinguishable from each other. Upon completion of the fourth trial, participants were asked whether they could identify the trial in which they received CM. Five participants correctly identified the CM trial, whereas eight participants were not able to correctly identify the CM trial, which suggests successful blinding of the intervention in this experiment.

### 2.5 Data treatment and statistical analysis

The primary outcome RSP was assessed by the sprint performance decrement (S_dec_; %), which quantifies fatigue by comparing actual sprint performance over the MST to ideal sprint performance, conceptualized as the fastest sprint time (FT) being repeated for each sprint (Fitzsimons et al., 1993; Glaister et al., 2008). The S_dec_ method is established as the most valid and reliable method of quantifying fatigue in tests of multiple sprint performance (Glaister et al., 2008). The following variables were recorded for analysis: fastest sprint time, the fastest sprint of each MST trial; total sprint time (TT), the sum of the 10 sprint times in one MST trial and from which average sprint time was calculated; and slowest sprint time (ST), the slowest sprint of each MST trial. S_dec_ (%) was calculated as: S_dec_ = {[TT/(FT x 10)] − 1} x 100. The smallest worthwhile difference (SWD) was set at 0.2*between‐subject standard deviation, which is suggested to represent a practically‐relevant change in performance in athletes. Thus, the SWD corresponded to 0.37% for S_dec_ in this study.

Statistical analyses were performed using Prism version 8.4 (GraphPad Software Inc). Data are reported as mean ± SD, or mean difference and 95% confidence interval (CI). Differences between CM and PLA for each variable were assessed by a paired samples *t*‐test, with the exception of heart rate and individual sprint times, which were assessed using a two‐way (condition *×* time) repeated measures analysis of variance (ANOVA). Effect sizes (ES) were calculated using Cohen's *d*. Statistical significance was accepted at the level of *p* < 0.05 for all statistical tests.

## 3 RESULTS

**FIGURE 2:**
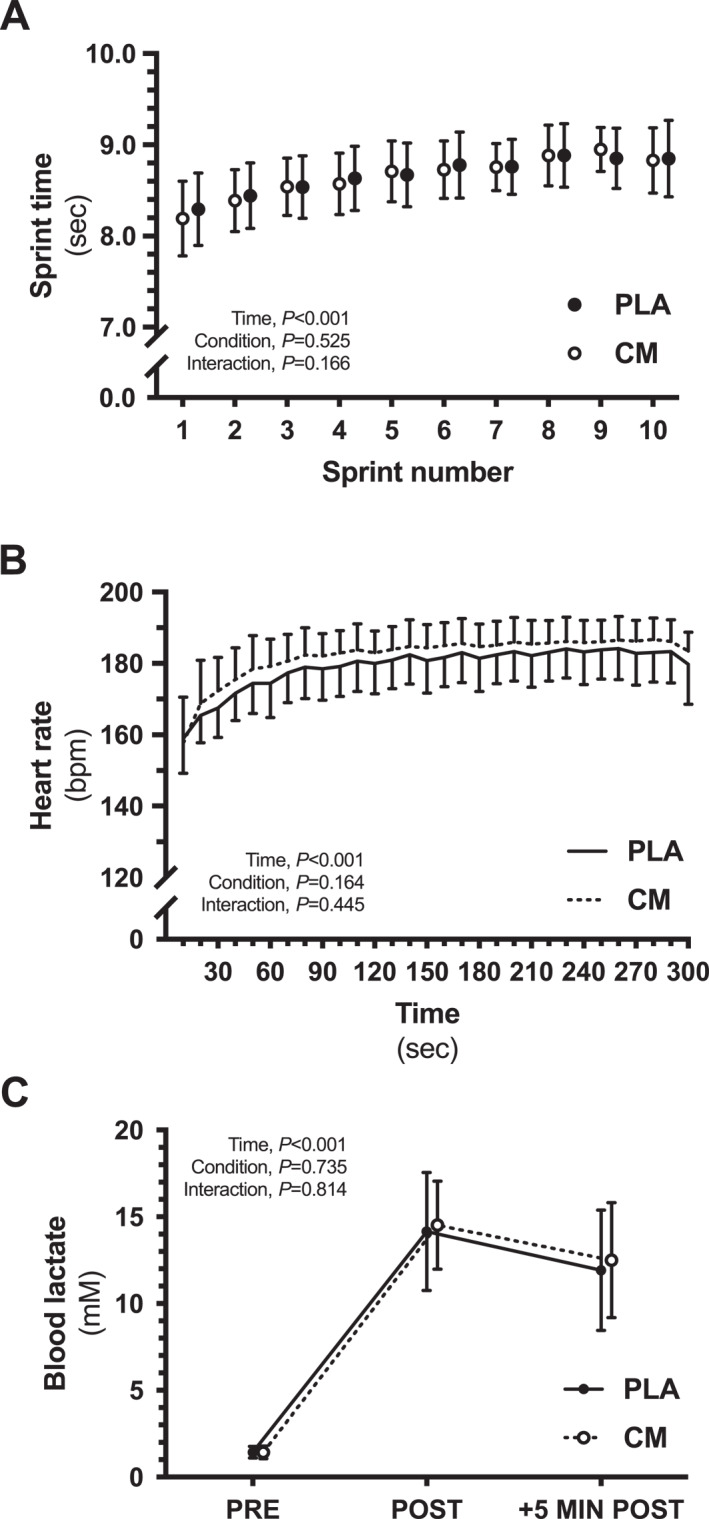
Sprint times over the individual sprints across the MST (A), heart rate during the MST (B), and blood lactate concentration, assessed before (PRE), immediately after (POST) and 5 min after (+5 min POST) completing the MST (C). Data are presented as mean values, with error bars representing standard deviation (*n* = 13). CM, citrulline malate; MST, 40 m maximal shuttle run test; PLA, placebo.

Blood lactate concentration, assessed before, immediately after, and 5 min after completing the MST, did not present interaction (*p* = 0.81) or condition effects (*p* = 0.73), but there was a main effect of time (*p* < 0.01) (Figure 2A). Similarly, heart rate (Figure 2B) and individual sprint time across the MST (Figure 2C) did not present interaction (*p* = 0.44 and *p* = 0.16, respectively) or condition effects (*p* = 0.16 and *p* = 0.52, respectively), but there was a main effect of time (*p* < 0.01 and *p* < 0.01, respectively).

**FIGURE 3:**
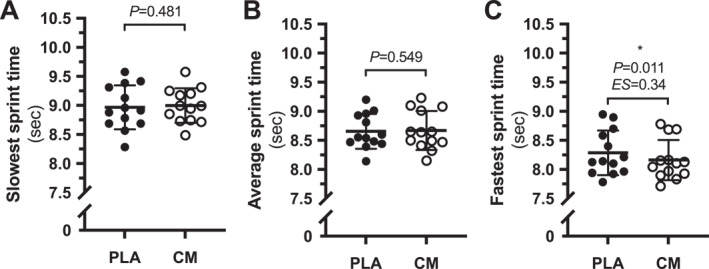
FT (A), average sprint time (B), and ST (C) during the MST. Data are presented as mean values, with error bars representing standard deviation (*n* = 13). *p = 0.011 and ES = 0.34 for FT between PLA and CM. CM, citrulline malate; FT, fastest sprint time; PLA, placebo; ST, slowest sprint time.

There was an improvement in the FT in CM compared to PLA (CM, 8.16 ± 0.34 s vs. PLA, 8.29 ± 0.39 s; *p* = 0.011; ES = 0.34) (Figure 3A). There were no differences between CM and PLA for average sprint time (CM, 8.67 ± 0.33 s vs. PLA, 8.65 ± 0.30 s; *p* = 0.54) (Figure 3B) or ST (CM, 8.99 ± 0.29 s vs. PLA, 8.96 ± 0.37 s; *p* = 0.48) (Figure 3C).

**FIGURE 4:**
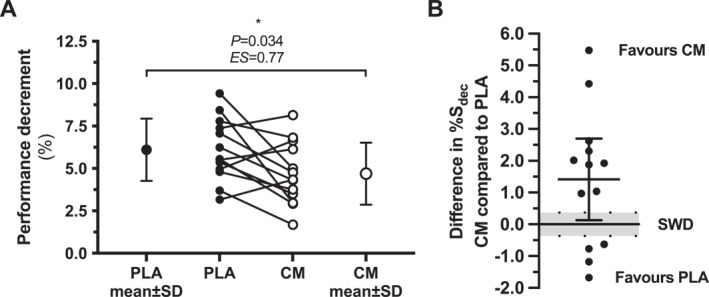
Performance decrement (A), and mean difference in % S_dec_ (B) during each trial. Data are presented as mean values, with error bars representing standard deviation (*n* = 13). *p = 0.034 and ES = 0.77 for performance decrement between PLA and CM. The shaded area in (B) represents the range for the smallest worthwhile difference in S_dec_ in this cohort, and the short horizontal line and error bars represent mean difference and 95% CI, respectively, comparing CM to PLA. S_dec_: sprint performance decrement; CM, citrulline malate; PLA, placebo.

RSP was improved (i.e., evidenced by a decrease S_dec_) in CM compared to PLA (S_dec_: CM, 4.68 ± 1.82% vs. PLA, 6.10 ± 1.83%; *p* = 0.034; ES = 0.77) (Figure 4A). The mean difference (95% CI) in S_dec_ between CM and PLA was 1.41% (0.13%–2.70%) (Figure 4B). Compared to PLA, nine participants demonstrated improvements in performance with CM that were greater than the SWD, whereas four participants demonstrated a decrement in performance with CM that was greater than SWD (Figure 4B).

## 4 DISCUSSION

In this study investigating the effect of 3 days of CM supplementation (8 g daily) on short‐duration high intensity exercise performance in male university‐level team sport athletes, running RSP, as measured by the sprint performance decrement, was improved by CM supplementation compared with PLA, without effects on blood lactate concentrations or heart rate.

The ergogenic effects of CM supplementation have been equivocal to date. A series of studies have reported no effects of CM on short‐duration exercise performance (Bezuglov et al., 2022; Chappell et al., 2020, 2018; Cunniffe et al., 2016; Farney et al., 2019; Gonzalez et al., 2018; Trexler, Keith, et al., 2019; Wax et al., 2015), while others have documented ergogenic effects (Glenn et al., 2017; Pérez‐Guisado & Jakeman, 2010; Wax et al., 2016). For example, CM (8 g dextrose + 8 g CM) supplemented 1 h before a resistance exercise protocol in 15 trained females resulted in an improvement in upper‐ and lower‐body performance measured by number of repetitions performed (Glenn et al., 2017). Similar enhancement in the number of repetitions performed during resistance exercise sessions has been observed in male participants provided with 8 g of CM 1 h prior to exercise (Pérez‐Guisado & Jakeman, 2010; Wax et al., 2016).

Yet prior to the results of the present study there was little evidence for the beneficial effects of L‐citrulline or CM on RSP. One week of L‐citrulline supplementation (6 g per day) did not improve RSP (6 × 1 min sprints at 120% of maximal power) performed after a 40 km time trial in trained cyclists (Stanelle et al., 2020), whereas CM supplementation (12 g) did not improve RSP (10 × 15 s sprints) in well‐trained men (Cunniffe et al., 2016). In contrast, the present study employed a short‐duration (∼5 min) high intensity exercise test of running RSP and observed a performance benefit in these team sport athletes. Direct comparison of previous studies to the present study is difficult because a variety of protocols (number of bouts/sprints/sessions/repetitions and work‐to‐rest ratios), exercise modalities (resistance exercise training, cycling, and running), supplement administration methods (dose, time before commencing exercise protocol, citrulline/malate ratios), and population of interest (male/female, fitness status, healthy or diseased) have been implemented. Hence, more studies must be conducted to increase the evidence‐base for CM as an ergogenic aid, and to clarify the mechanisms of action under different conditions.

Despite the overall finding of a positive effect of CM on RSP, four participants (∼30%) had decrements in performance after CM supplementation when compared to PLA that were greater than the SWD calculated for this test and cohort. Such observations could be explained by the inter‐individual response to supplement use (Burke, 2017), and the influence of factors such as sex, genetic differences, and training status. In practical terms, this suggests that individual athletes should, where possible, test the ergogenic potential of CM for themselves in an ecologically‐valid test of their own performance. Regarding training status, highly‐trained and elite athletes may respond differently to some sports nutrition strategies compared to their lesser‐trained or less successful counterparts (Burke, 2017), with one such example being nitrate supplementation being less effective in individuals with greater aerobic fitness (Porcelli et al., 2015). Given that the mechanisms of ergogenic action of L‐citrulline and nitrate are related, it is possible that ergogenic effects of CM could be similarly influenced by aerobic fitness or training status. A limitation of the present study, therefore, is that we did not assess the aerobic fitness of our participants.

The mechanisms of ergogenic action of CM are proposed around the respective effects of the citrulline and malate molecules. Firstly, the citrulline component via the L‐arginine NO pathway (Vanhoutte et al., 2016) may improve the delivery of blood (and oxygen) to and from the active musculature during exercise through vasodilatory properties (Gonzalez & Trexler, 2020; Gough et al., 2021). Secondly, the citrulline component may assist in ammonia detoxification through the urea cycle, decrease lactate production, and enhance the erobic utilization of pyruvate, thereby improving muscle function and attenuating fatigue (Gonzalez & Trexler, 2020). Thirdly, the malate component may act as a TCA cycle intermediate, which has been proposed to augment energy production and increase the rate of ATP production (Bendahan et al., 2002; Gonzalez & Trexler, 2020). One of the most critical controls of the rate of aerobic ATP production is oxaloacetate, and because malate is dehydrogenated into this compound in the TCA cycle, it may offer an explanation to the purported additive effects of CM over L‐citrulline alone (Bendahan et al., 2002). Hence, citrulline and malate may work synergistically to increase skeletal muscle tissue perfusion and enhance the efficiency of ATP production to improve exercise performance (Gonzalez & Trexler, 2020).

In the present study, like most studies in this field, assessment of these proposed mechanisms is limited and indirect at best. Blood lactate concentrations and heart rate, as surrogates of energy metabolism and cardiovascular responses, respectively, were not affected by CM supplementation, which is consistent with previous findings for lactate concentration (Bezuglov et al., 2022; Chappell et al., 2020; Farney et al., 2019; Rhim et al., 2020; Trexler, Keith, et al., 2019; Wax et al., 2015; Wax et al., 2016) and heart rate (Farney et al., 2019; Glenn et al., 2017; Wax et al., 2015; Wax et al., 2016). These measures are considered as indicators of the intensity of exercise and whole‐body physiological stress, but do not provide any mechanistic insight into ergogenicity of CM. Future studies would be needed to replicate our initial findings, and to interrogate mechanisms of action in order to improve the understanding on the effects of CM on short‐duration high‐intensity exercise performance.

Strengths of the experimental protocol include employing a placebo‐controlled, double‐blind, counterbalanced cross‐over design, and the standardization of participant preparation, time of day, and the testing environment, yet some limitations must be acknowledged. Firstly, as mentioned above, fitness status was not assessed, but in mitigation, an important inclusion criterion was that only athletes actively training and competing at least three times weekly in a team sport could participate. Secondly, we employed a convenience sampling approach within the time constraints of an academic semester resulting in recruitment of only male sport team athletes and a sample size of 13. Repeating this study in female participants would be relevant in order to understand the influence of CM on performance in female team sport athletes, and improve the generalizability of the findings. Although we observed a benefit of CM on RSP with the sample size of 13, it is arguable that this represents an underpowered analysis. Using an assumed effect size of 0.2 (“small”) based on the data from a number of meta‐analyses (Trexler, Persky, et al., 2019; Vårvik et al., 2021; Viribay et al., 2022), the required sample size for a two condition cross‐over design is ∼200 based on an alpha level of 0.05 and a beta level of 0.2, whereas a *n* size of 13 would have required the assumed effect size to be 0.85 (“large”) (G*Power v3.1). Lastly, a related point is that there was no condition × time interaction effect for the individual sprint times, despite the finding of a significant difference between conditions for S_dec_. This absence of an interaction effect may reflect that the study would also be underpowered for that ANOVA analysis, and result in a type II error. However, because the S_dec_ calculation includes the FT as the key denominator, we cannot exclude the fact that the difference in S_dec_ between CM and PLA is largely explained by the FT being better in CM, and thereby proportionately influencing the S_dec_ calculation.

In conclusion, the present study observed that 3 days of 8 g daily of CM supplementation attenuated the sprint performance decrement that occurred during a 10 repetition 40 m MST employed as a test of short‐duration (5 min) high‐intensity exercise performance in male university‐level team sport athletes. Indeed, further studies are needed to explore the mechanisms of possible ergogenic action of CM on RSP, and to perform similar studies in female athletes. The outcomes of this study suggest that short‐term CM supplementation could be beneficial for team sport athletes who perform short‐duration high‐intensity efforts, such as soccer, Gaelic games, field hockey, and rugby.

## CONFLICT OF INTEREST STATEMENT

No conflict of interest, financial or otherwise, is declared by the authors.
